# Safety Evaluation in Healthy Colombian Volunteers of P2Et Extract Obtained from *Caesalpinia spinosa*: Design 3+3 Phase I Clinical Trial

**DOI:** 10.1155/2022/7943001

**Published:** 2022-02-23

**Authors:** María I Duran, Ricardo Ballesteros-Ramírez, Angélica Tellez, Lilian Torregrosa, Peter A Olejua, Silvia Galvis, Claudia Urueña, Susana Fiorentino

**Affiliations:** ^1^Grupo de Inmunobiología y Biología Celular, Unidad de Investigación en Ciencias Biomédicas, Facultad de Ciencias, Pontificia Universidad Javeriana, Bogotá, Colombia; ^2^Departamento de Ciencias Fisiológicas, Facultad de Medicina, Pontificia Universidad Javeriana, Bogotá, Colombia; ^3^Hospital Universitario San Ignacio, Centro Javeriano de Oncología, Facultad de Medicina, Pontificia Universidad Javeriana, Bogotá, Colombia; ^4^Centro de Investigaciones, Hospital Universitario San Ignacio, Bogotá, Colombia

## Abstract

The polyphenol-enriched extract called P2Et derived from *Caesalpinia spinosa (C. spinosa)* had antitumor and immunomodulatory activities reported in breast cancer, leukemia, and melanoma. The aim of this study was to evaluate the safety and maximum tolerated dose of P2Et extract in Colombian healthy volunteers in a phase 1 clinical trial, open labelled, single-arm, dose-escalation design 3 + 3. Seven healthy volunteers were included; P2Et was administrated in capsules of 600 mg/d for 28 days. Analysis by intention to treat was performed. 4 volunteers showed adverse events and discontinued the intervention. 94.6% of AE were grade 1, and most of AE had a reasonable possibility of a relationship with the P2Et (83.8%). We found that the oral administration of P2Et is safe in healthy humans with a maximum tolerated dose of 600 mg/d. There was no severe toxicity; most of the adverse events were mild, without significant changes in the safety parameters evaluated.

## 1. Introduction

*C. spinosa* or Dividivi has been traditionally used by Colombian indigenous tribes located in the Caribbean coast. Its traditional medicinal uses include antimicrobial, antioxidant, antitumor, and immunomodulatory properties for different diseases [1–3].

P2Et has shown immunomodulatory and antitumoral properties [3–6]. The immunomodulatory effect of the P2Et seems to be dependent on the basal conditions of host. In healthy C57BL/6 mice treated twice a week for 21 days with the extract, an increase in the number of CD4+ and CD8+ activated T cells, NK cells, regulatory T cells, dendritic cells, and myeloid-derived suppressor cells (MDSC) in lymphoid organs was observed, together with a significant increase in the concentration of serum cytokines such as IL-10, IL-17, IFN-alpha, IL-6, IL-4, and IL-2 [4]. In contrast, when immunocompetent mice orthotopically transplanted with 4T1 tumoral cells (highly metastatic breast cancer cell line) were treated with P2Et, a decrease in primary tumor growth and metastatic foci was observed [4]. In a melanoma murine model, the P2Et induces immunogenic cell death favoring immune system activation, particularly of T cells, allowing generation of specific multifunctional memory T cells, IFN-*γ*, TNF-*α*, IL-4, and IL-5 producers which could participate in primary tumor control [7].

To ensure the safety of herbal pharmaceutical preparations, safety and tolerability studies are a fundamental requirement to access the clinical stages efficacy in humans. The P2Et has a potential to be developed as an efficient herbal drug for the coadjuvant treatment in cancer and diseases with an inflammatory component. Thus, the clinical phase I study was carried out to evaluate the effect of continuous administration of the extract for 28 days in humans.

## 2. Materials and Methods

### 2.1. Preclinical Toxicology Test

#### 2.1.1. Animals and Housing Conditions

The animals were housed in specific cages for the species (*Rattus norvegicus and Oryctolagus cuniculus*) in accordance with current legislation that provides integrity and animal welfare.

The rats (*Rattus norvegicus*) were kept in a specific room with a temperature between 17.0 and 20.6°C and relative humidity between 54.9 and 70.2%. The rabbits (*Oryctolagus cuniculus*) were kept in a specific room with a temperature between 17.4 and 20.8°C and relative humidity between 54.9 and 70.2%. Both species were subjected to a photoperiod of 12 hours of light and 12 hours of darkness. The feeding of the animals consisted of conventional food for the species and filtered drinking water. Periodic microbiological analyses were carried out on the water and food, guaranteeing that they were suitable for consumption and did not interfere in the results of the tests.

Animals were maintained in the test facilities according to local and international requirements, based on the Guide for the Care and Use of Laboratory Animals [8]. Animals showing continuing signs of severe distress and/or pain at any stage of the tests were euthanized. Procedures for animal care and criteria for making the decision to euthanize an animal were based on the Guidance Document on the Recognition, Assessment, and Use of Clinical Signs as Humane Endpoints for Experimental Animals Used in Safety Evaluation [9].

The chronic toxicity study was performed in a group of rats and rabbits in a GLP certified center. The study procedures were aligned to ICH M3 (R2) [10]. 12 rabbits of 11 weeks of age were used: 6 experimental animals and 6 control animals with equal number of males and females in each group (Table S1). 20 rats of 9 weeks of age were used for the test: 10 experimental animals and 10 control animals with equal number of males and females in each group (Table S1).

The P2Et was administered at the dose of 1000 mg/kg by oral route once a day, for a period of 28 days, by oral gavage with syringe and gastric tube. Oral administration was chosen due to the intended indicated use of the product.

#### 2.1.2. Dose Calculation

The starting dose of P2Et for the clinical trial in humans was estimated based on the preclinical data and NOAEL of P2Et in the repeated chronic toxicity study according to FDA Guidance for Industry-Estimating the Maximum Safe Starting Dose in Initial Clinical Trials for Therapeutics in Adult Healthy Volunteers [11].

#### 2.1.3. Statistical Analysis

Toxicity tests were analyzed using BioEstat 5.3; for parametric data, t-student test was used for comparing between experimental and control groups, and Mann-Whitney test was employed for nonparametric data. The significance level used was *p* < 0.05.

### 2.2. Clinical Study

#### 2.2.1. Study Volunteers

The clinical study was conducted aligned to Declaration of Helsinki, Good Clinical Practice (GCP), and local regulation [12–14]. The study was reviewed and approved by the independent ethics committee (IEC) of the Hospital Universitario San Ignacio. Trial was registered in clinical trial no. NCT03663881 (https://clinicaltrials.gov/ct2/show/NCT03663881). This is a single-centered clinical trial conducted in the Office of Research of the Hospital Universitario San Ignacio. The subjects were recruited from hospital workers, students, and relatives of patients from different departments of the hospital. All subjects read and signed the informed consent form before performing any study procedure. Subjects were evaluated for eligibility through a medical history, vital signs, physical examination, clinical laboratory tests, and electrocardiogram (ECG).

Following were inclusion criteria: participants should be healthy, between 18 to 65 years old, without significant comorbidities, and with adequate hepatic, renal, and hematological function. Additionally, eligible female subjects are not pregnant or lactating and if they were of childbearing age, they must be using at least one effective contraceptive method. The exclusion criteria were the following: (1) active disease or infection such as HIV positive or history of severe hepatic, cardiac, pulmonary, endocrine, or psychiatric morbidity; (2) history of allergic reactions to polyphenol-type compounds; (3) use of dietary supplements or phytomedicines <15 days prior to entry and during the study or strong or moderate inducers and inhibitors of CYP3A 7 days prior to the start of study treatment; (4) history of drug or alcohol abuse or use of an investigational product and/or participation in another clinical study.

#### 2.2.2. Study Design

**Figure 1:**
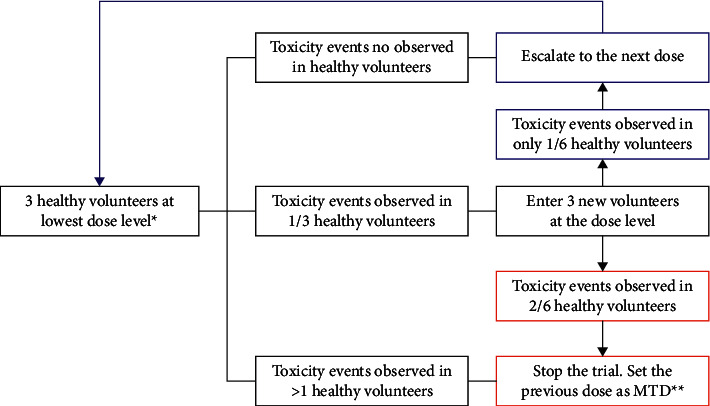
Clinical trial design 3 + 3 for the P2Et in healthy volunteers. ^*∗*^The lowest dose in this trial is 600 mg/d for 28 days. ^*∗∗*^MTD: maximum tolerated dose (modified from [15]).

This study was open label, single arm, representing a phase 1 study for the safety evaluation of the P2Et in healthy subjects, with dose escalation to establish the maximum tolerated dose (MDT) and toxicity at dose limits (TDL). A classic 3 + 3 design was used for dose escalation (Figure 1).

Dosage escalation of P2Et was planned with 4 dosage levels: 600 mg, 1200 mg, 2400 mg, and 4800 mg per day. At least 3 different subjects would have to be enrolled at each dose level. If TDL was not observed in the first 3 subjects treated at 28 days, they would proceed to escalation to the next dose level. If one or more TDL occurred at any dose level in a subject, 3 subjects would be added to that cohort. Any dose level at which 2 out of 6 subjects experienced a TDL was considered intolerable, and MDT would be considered if escalation was discontinued. In the case that the MDT was observed in the lowest dose level, and according to the clinical toxicity events, this could be set as the MDT.

#### 2.2.3. Study Medication/Herbal Extract

**Figure 2:**
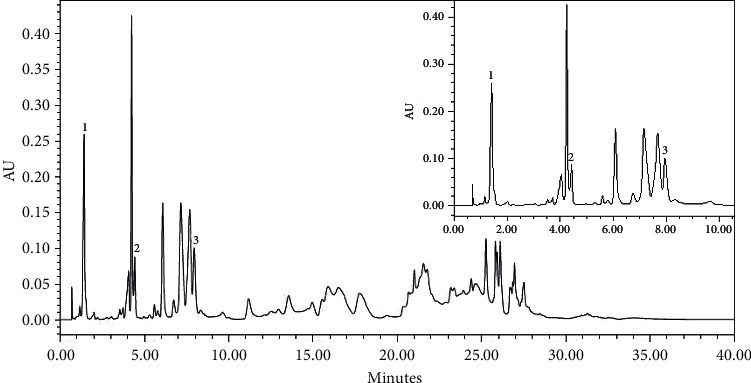
UPLC-PDA chromatogram at 274 nm of P2Et extract. Peak identification: (1) gallic acid; (2) methyl gallate; (3) ethyl gallate.

Pods of *C. spinosa* (Feuillée ex Molina) Kuntze (Divi-divi or tara) were collected in Villa de Leyva, Boyacá, Colombia, and identified by Carlos Alberto Parra of the National Herbarium of Colombia (copy number of voucher COL 588448). The P2Et is produced as described from the pods of *C. spinosa* [1, 16]. Briefly, fresh pods from *C. spinosa* were dried under airflow in a solar oven at 35°C and ground down to obtain plant material dried. This plant material was certified according to FDA Guidelines for Herbal Medicinal Products. Subsequently, the plant material was extracted with ethanol (96%) in a recirculating percolator. The ethanol crude extract was concentrated under vacuum and trapped on silica gel, and excess humidity was removed at 25°C. Afterward, the ethanol extract was fractionated with ethyl acetate. This herbal drug is manufactured in a validated production process under GMP conditions in LABFARVE laboratories. The standardization of the extract was carried out according to the FDA Guidance for Herbal Drugs [17]. For the standardization, multiple compounds derived from gallic acid were identified such as ethyl gallate, methyl gallate, ethyl 4,5-digaloyl quinate, methyl 4,5-digaloyl quinate, ethyl 3,5-digaloyl quinate, 4,5- digaloylquinic, ethyl 3,4,5-trigaloyl quinate, ethyl 5-galloyl quinate, among others [5] (Figure 2). Three main compounds were used for the normalization of P2Et: Ethyl gallate, methyl gallate, and gallic acid. Ethyl gallate is between 71.45 and 153.49 ug/mL, methyl gallate is between 7.89 and 27.30 ug/mL, and gallic acid is between 11.34 and 98.54 ug/mL. Also, the cytotoxic activity is measured in 4T1 and MCF-7 cell lines, where the CI50 could not be higher than 50,81 ug/mL and 132,75 ug/mL for each cell line, respectively.

With these specifications and the standardized production, qualitative and quantitative identification of these molecules is carried out in each batch produced, guaranteeing high batch-to-batch consistency for P2Et. The studies of natural and accelerated stability of P2Et were carried out according to the ICH and WHO regulations for zone IVB [10], where the P2Et is stable for at least 12 months, maintaining chemical and therapeutic integrity. To start producing a batch, plant material and P2Et must meet quality controls established by regulatory guide 2266 of 2004 of herbal drugs in Colombia, article 561 from USP 34 NF 29, and WHO guideline “*Quality Control Methods for Herbal Materials”* and FDA guideline “*Botanical Drug Development Guidance for Industry*.”

In this trial, the P2Et was used in a dosage capsule of 600 mg of active ingredient with starch as excipient to be taken daily and manufactured in LABFARVE laboratories under GMP conditions.

The presentation of extract was a bottle with 28 capsules, with a label according to requirements of the local regulation for clinical trials. The investigational product was stored in the research center from Hospital Universitario San Ignacio at a temperature below 30°C and humidity below 70.0 guaranteeing the stability.

#### 2.2.4. Tolerability and Safety Assessment

Adverse events, tolerability, and safety were evaluated based on the National Institute of Health Common Terminology Criteria for Adverse Events (CTCAE) version 4.0 [18] at the visit day 1 or baseline, day 8, day 15, and day 28, or final visit, which included clinical follow-up, measuring vital signs (pulse, blood pressure), and physical examination (including weight and height). The clinical laboratory tests were performed at baseline and final visit in the laboratory of the Hospital Universitario San Ignacio. The safety assessments included kidney, hepatic, electrolyte, metabolic, and hematological parameters (Table 1).

All adverse events that occurred during the trial were recorded along with the duration, severity, and relationship to the P2Et. TDL was considered as any grade 3 or greater hematological and nonhematological toxicity, observed during the 28 days of treatment according to CTCAE version 4.0. The tolerability and safety profile were assessed by the clinical team; they classified the laboratories out of range and if they had clinical and safety implications on the volunteers.

The evaluation of immunological parameters was included given background of the P2Et, which was assessed immunophenotype in peripheral blood, protein electrophoresis, and level of IgG, IgM, and IgA antibodies and complement (C3) protein.

#### 2.2.5. Statistical Analysis

An intention-to-treat analysis was performed using SPSS, version 23. Descriptive analysis methods were used for demographic data and vital signs. The frequencies and percentages for the analysis of adverse events were recorded. Data from the safety and immunological laboratories were analyzed with mean, standard deviation, in some cases median with ranges, and the percentage of change from the baseline visit to completion. *p* < 0.05 is considered significant.

## 3. Results

### 3.1. Preclinical Toxicity Results

There was no statistical difference between initial and final weights and food intake in rats and rabbits between the groups (Table S1). Also, there was no difference in the absolute and relative weights of liver, spleen, and kidneys in both groups. The analysis of the results of hemogram (erythrogram and leucogram), coagulogram, and kidney function indicate the absence of relevant alterations in rats and rabbits (Tables 2–3). There were no systemic clinical signs of toxicity during 28 days of observation, and macroscopic findings in the necropsy were not observed between groups. These results showed that no observed adverse effect's level (NOAEL) for P2Et is 1000 mg/kg for rabbits and rats.

The rabbit's weight was used for the calculation of the starting dose in the clinical trial as we did not find differences between rabbits and rats; we selected rabbits as superior specie. Once a starting dose level has been determined, other levels of doses were duplicates. The dose calculated was 600 mg/d for 28 days in the clinical trial.

The P2Et was previously assessed for genotoxicity and mutagenicity activity in a previous study. It did not show genotoxicity and mutagenicity activity [16].

### 3.2. Clinical Results

#### 3.2.1. Subject Characteristics

Eleven subjects were screened, of which 7 were enrolled in the study at the first dosage level. 3 subjects completed 600 mg/d level and the remainder were discontinued from the study due to adverse events with a reasonable possibility of relationship to the investigational product.

The maximum sample size planned was between 12 and 24 if we could scale the doses level, but TDL was observed at the first dose level; 4 volunteers showed adverse events and discontinued the intervention; considering that at any dose level at which at least 2 subjects experienced TDL was considered intolerable, we set the MDT at the first dose level (600 mg/d).  Table 4 summarizes the main baseline characteristics of the subjects. The range of exposure to the investigational product was between 2 and 28 days. The adherence during the exposure period was greater than 80%.

#### 3.2.2. Safety

The MTD of the P2Et was 600 mg/d. In the first cohort of 3 subjects, 1 presented TDL requiring the inclusion of 3 additional subjects, of which 2 subjects presented TDL. The TDL reported in these 3 subjects was defined by the investigator since the adverse events were clinically significant and led to the discontinuation of these study subjects.

A total of 42 nonserious adverse events (AEs) were reported; the majority of AEs were grade 1 in severity (95%) according to CTCAE and a reasonable possibility of a relationship with the P2Et. There were no serious adverse events (SAEs) in the subjects participating in the clinical study. The most frequent AEs were gastrointestinal events such as epigastralgia and nausea in 23.8%; only one subject had a slight increase in AST who recovered without sequelae. The detail of the incidence of reported adverse events is described in Table 5.

No changes outside the normal range were observed from baseline to final visit in vital signs and physical examination in all subjects. Cardiovascular function did not present any alteration in most of the subjects, except for one subject who presented an abnormality in the electrocardiogram with a marked sinus bradycardia, incomplete right bundle branch block, and left fascicular block since the baseline visit, being classified as clinically not significant and considered as a medical history.

The hematological parameters evaluated were within normal ranges at the initial and final visit, except for LDH, which was found with a median below the lower normal limit, as well as the mean platelet volume (MPV). In the evaluation of renal function, no changes outside the normal range were observed, only a slight increase in the upper limit of the range of the median blood urea nitrogen at the initial and final visit. Likewise, hepatic, electrolyte, and metabolic function remained within normal ranges; only the AST level at the completion visit showed a slight increase in the upper limit of the median range. Table 1 shows the changes in blood chemistry markers evaluated at the initial and final visit.

#### 3.2.3. Immunological Parameters

As seen in Table 6, the results of the peripheral blood immunophenotype for enrolled subjects since baselines to end visit showed a median frequency of LT CD3^+^/CD5^+^/CD45^+^, CD3^+^/CD4^+^, and CD3^+^/CD8^+^ below the normal lower limit at both times. The above was not observed in the absolute count of these cell populations.

The count of LT CD4^+^/CD8^+^ presented a 40% of median of percentage of change between baseline and final visit; relation CD4/CD8 was preserved. Population of B lymphocytes (LB) CD19^+^/CD20^+^/CD38^+^/CD45^+^ had a median below the normal lower limit in basal and final visit, with a percentage of change lower than 20%. Additionally, a percentage of positive change from the initial to final visit of 66% was observed in the median percentage of monocyte-derived dendritic cells CD45 ^++^/CD38 ^+^ /CD4 ^+^. Other cell populations assessed were within the normal range.

In protein electrophoresis, the mean of the alpha 1 fraction remained outside the normal range (1% to 2.8%) between baseline ((3.49%) and final visit (3.84%), with a percentage change of 10.6%. The levels of the different immunoglobulins evaluated and the level of complement were in normal limits in both visits.

## 4. Discussion

Polyphenols have been used in healthy humans for the prevention of diseases such as cancer, cardiovascular disease, diabetes, neurological disorders, infections, among others. The above, given its antioxidant, anti-inflammatory, antiallergy, and even immunomodulatory effects [19–26], is also useful for the treatment of some respiratory diseases [27].

Although its potential for use is extensive, the side effects generated by the consumption of natural products prepared in the form of standardized extracts have not been systematically evaluated. The development of formal clinical studies makes it possible to strengthen knowledge on dosage, population characteristics, and possible adverse events and opens the possibility of advancing in the evaluation of therapeutic effects in patients with different pathologies.

In the preclinical toxicology study, no significant differences were observed between the experimental and the control group that were associated with clinical signs of toxicities. Despite the use of the *Caesalpinia spinosa* in different traditional preparations, there are no reports of oral toxicity studies evaluating the safety of these preparations or standardized extracts. These results allow us to obtain the NOAEL for P2Et at 28 days in continuous administration; this value is 1000 mg/Kg. The NOAEL for other toxicity studies of polyphenolic compounds obtained from green tea show NOAEL values slightly lower than that found for P2Et [28].

As a result of the preclinical toxicology study, 600 mg/d was defined as the MDT of the P2Et in healthy subjects. It was evidenced that gastrointestinal AEs were the main AEs associated with the consumption of P2Et with an incidence of 52% (*n* = 22/42); the most frequent event was epigastric pain, nausea, constipation, reflux, abdominal distention, and flatulence, with nonserious characteristics, severity grade 1, and 85% with reasonable possibility related to the product. In the same way, other phase 1 clinical trials [29–33] have reported this type of adverse events such as nausea, diarrhea, and mild abdominal pain, with a frequency of 16% to 91% with the use of other polyphenols (turmeric, epigallocatechin gallate, resveratrol, and sahastara) in healthy humans; the above could have an impact on adherence and quality of life of people due to adverse event severity.

In addition to the above, other types of adverse events were reported in various functional systems in 48% (*n* = 20/42) such as myalgia, asthenia, adynamia, and respiratory symptoms (odynophagia, rhinopharyngitis, and cough). Likewise, dermatological and central nervous system adverse events such as pruritus, dermatitis, dizziness, headache, and drowsiness were reported in other clinical studies of polyphenols in healthy subjects with a frequency greater than 30%, which seem to be signs of toxicity that they are not normally associated with this type of natural product [29, 32, 34–36]. Thus, there is great diversity in the adverse events that polyphenols can cause in healthy humans, which may be associated with their route of administration, duration of consumption, and the metabolism of each of the polyphenols.

The analysis of hepatic function parameters did not show significant alterations, only one outside the normal range at the limit of the maximum range of the median in the AST level, taking into account that only one subject had an AST increase during the study that was resolved without complication and sequel.

Alterations in hepatic function are the most severely monitored toxic effects that can occur in healthy people who consume polyphenols or phytomedicines, even without having risk factors for hepatic disease. In recent years, a significant increase in cases of hepatic damage attributed to the phenomenon of herbal supplements has been reported, being the second most frequent (16.53%) after antibiotics to cause drug-induced liver injury in the United States [37]. In fact, these hepatic abnormalities are reflected in increased levels of total bilirubin, AST, and alkaline phosphatase [29, 33], most of them associated with the use of high doses of resveratrol and *Bacopa monnieri* and self-limited with the discontinuation of their consumption. Therefore, hepatic toxicity seems to be dose dependent and although it is not the most frequent alteration, given the severity of its appearance, it must always be monitored and taken into account for the safety evaluation after the consumption of polyphenols in healthy humans.

In the same way, kidney function can be affected by the consumption of polyphenols since during the metabolism some of its products can generate kidney damage; however, in this study only levels of urea nitrogen were evidenced outside the normal range in the limit of the maximum range of the median, which was not considered significant. There are some intrinsic factors that can increase the risk of kidney injury, such as the quality of polyphenol manufacture, overdose, interactions with other medications, and pathological conditions of the individual, including gender and age [38]. For example, increased levels of urea nitrogen and creatinine have been linked to the use of high doses of polyphenols such as sahastara, *Crocus sativus*, and *Bacopa monnieri* [32, 33, 39]. Other polyphenols have not shown alteration in kidney function or in some studies it is unknown because it was not evaluated [29–31, 39–43]. Thus, hepatic toxicity and kidney alterations, although they are not very frequent and depend on other factors, should be closely monitored when using this kind of products.

The mean of the metabolic parameters evaluated in this study was within the normal range, with the exception of total cholesterol, which was reported slightly outside the maximum range of the median from the initial visit persisting until the end visit, without a significant change percentage. Similarly, there was a positive percentage change of 18% in median HDL from baseline to final visit. Given the effects on the prevention of cardiovascular disease of some polyphenols, there are studies that report a benefit such as a decrease in total cholesterol and triglycerides and an increase in HDL [25, 32]. In contrast, some clinical studies have not reported changes in lipid profile [39, 42–44] and even negative effects such as that high doses of carvacrol induced a decrease in HDL and an increase in triglycerides [34]. Thus, the data associated with the possible effect on lipid metabolism of polyphenols in healthy subjects are not conclusive and are divergent, suggesting that the differences, not only in these parameters, but in those previously mentioned, may be due to molecular diversity of polyphenols, as well as the variations inherent in the populations studied.

Now, part of the secondary endpoints of the study was the evaluation of immunological parameters, taking into account the preclinical history of immunomodulation of the P2Et. The quantification of the lymphoid and myeloid population remained within normal ranges, as were the levels of IgM, IgA, IgG, and complement C3 protein, with the exception of the percentage of LT CD3 + /CD5 + /CD45 +, CD3 + /CD4 +, CD3 + /CD8 +, and LB CD19 + /CD20/CD38 + /CD45 + that were reported below the lower normal limit from the baseline to final visit, with percentages of positive change less than 30%. It is possible that the normal values of our population are different from those currently used by the clinical reference laboratory.

Additionally, there was a 66% positive change percentage from the initial to final visit in the median percentage of monocyte-derived dendritic cells CD45 ++/CD38 + /CD4 +. In the same way, the absolute value of these cells also showed an increase from the baseline to the end visit with a change percentage of 34%. The aforementioned findings were not clinically significant, but they show a considerable increase in the dendritic cell population when the P2Et is consumed. These findings could suggest an increase in the production of their precursors in the bone marrow, in the generation of DCs derived from monocytes, or in their maturation; however we do not know the real origin of this increase and its impact on the functionality of these cells after the use of P2Et. However, this immunostimulatory effect of increasing antigen-presenting cells in healthy humans could be associated with a state of readiness for a more effective immune response in case of exposure to antigens. In the case of P2Et, congruence between preclinical data in animals and clinical data in healthy subjects is evidenced, confirming its immunomodulatory effect. In fact the P2Et has shown antitumor and immunomodulatory activity in some types of cancer such as breast cancer, leukemia, and melanoma [1,3–7, 45] and its administration also modulates the immune response of healthy mice [4]. In humans, it is still controversial. It is possible that the effects of certain polyphenols on the immune system may depend on the type of polyphenol, its dosage, and exposure time. There are other polyphenols for which their immunomodulatory activity has been studied in healthy people, finding a significant decrease in total LT, LT CD8 +, and NK [46]; or on the contrary, others present an immunostimulatory effect with an increase in cytotoxic LT, NK, and LB [47–49].

## 5. Conclusions

The P2Et consumed was safe for use in healthy humans with a maximum tolerated dose of 600 mg/d. There was no severe toxicity, only mild adverse events for the most part, with no significant out-of-range changes in the safety parameters evaluated. Changes in immunological parameters were observed that could suggest the immunomodulatory activity of P2Et also observed in animal models. Levels higher than 600 mg/d were not reached because of the gastrointestinal events showed for the volunteers at the first dose level. Despite the number of recruited volunteers, the MDT was set at 600 mg/d according to the 3 + 3 design for clinical trials. The foregoing will allow continuing with the clinical development of the extract given its preclinical history; it could be in a population with cancer, diseases with an immunological component, and even infectious diseases.

## Figures and Tables

**Table 1:** Safety assessments: kidney, hepatic, electrolyte, metabolic, and hematological parameters.

	Extract P2Et 600 mg/d		
	Baseline visit	End visit	% Change
	(*n* = 7)	(*n* = 7)	
Urea nitrogen blood (mg/dL)	13.7 [11.1, 20.4]	13.0 [9.10, 23.7]	2.36 [−20.2, 73.0]
Serum creatinine (mg/dL)	0.870 [0.630, 1.09]	0.860 [0.650, 1.14]	−1.15 [−9.09, 10.7]
AST (U/L)	20.0 [12.0, 22.0]	22.0 [13.0, 50.0]	8.33 [−11.1, 127]
ALT (U/L)	19.0 [14.0, 23.0]	21.0 [14.0, 44.0]	13.3 [−21.1, 214]
Total bilirubin (mg/dL)	0.690 [0.470, 1.08]	0.660 [0.420, 0.920]	−8.33 [−28.6, 58.6]
Sodium (mmol/L)	139 [136, 141]	138 [136, 141]	0.00 [−2.16, 0.735]
Potassium (mmol/L)	4.40 [4.00, 4.80]	4.50 [4.00, 5.00]	0.00 [−6.98, 12.5]
Calcium (mg/dL)	9.50 [9.10, 9.70]	9.50 [9.00, 9.70]	0.00 [−5.26, 4.40]
Magnesium (mg/dL)	2.00 [1.80, 2.10]	2.00 [1.80, 2.10]	0.00 [−5.26, 11.1]
Plasma glucose (mg/dl)	84.0 [78.0, 99.0]	88.0 [78.0, 96.0]	1.18 [−3.03, 4.76]
Total cholesterol (mg/dl)	170 [122, 250]	178 [98.7, 226]	−3.47 [−19.4, 4.66]
Triglycerides (mg/dl)	69.7 [47.7, 147]	70.2 [40.4, 161]	−6.33 [−48.7, 84.9]
Sht (Uu/mL)	1.24 [0.690, 3.42]	1.44 [0.630, 2.09]	−8.70 [−38.9, 55.6]
C-reactive protein (mg/dl)	0.100 [0.03, 0.50]	0.180 [0.05, 2.17]	133 [−64.0, 570]
Lactate dehydrogenase (U/L)	159 [130, 174]	161 [128, 210]	3.12 [−4.47, 31.8]
Hemoglobin (g/dl)	14.7 [13.6, 17.9]	14.5 [14.1, 16.7]	0.621 [−6.70, 6.62]
Hematocrit (%)	43.0 [40.4, 51.0]	43.1 [41.0, 47.9]	1.05 [−7.06, 5.12]
Medium corpuscular volume (fL)	89.0 [87.1, 97.2]	88.3 [87.4, 98.2]	0.344 [−2.97, 1.03]
Mean corpuscular hemoglobin (pg)	30.5 [29.6, 33.3]	30.6 [29.7, 33.8]	0.338 [−1.98, 1.50]
Mean corpuscular hemoglobin concentration (g/dl)	34.0 [33.5, 35.1]	34.1 [33.1, 35.2]	0.285 [−2.93, 2.67]
MPV (fL)	7.10 [6.90, 9.20]	7.50 [6.70, 9.00]	1.35 [−3.80, 7.04]
Erythrocyte sedimentation rate (mm/h)	2.00 [2.00, 10.0]	5.00 [2.00, 16.0]	0.00 [−50.0, 700]

Data are in median [minimum, maximum] range; (*n* = 7). AST, aspartate aminotransferase; ALT, alanine aminotransferase; SHT, stimulating hormone thyroid; MPV, medium platelet volume.

**Table 2:** Effects of P2Et extract on biochemical parameters and hematological parameters in rats following 28 days of administration.

	Parameters (reference values)	Group		*p* value
		Test (N = 10)	Control (N = 10)	
Kidney function	Urea (18.0 to 45.0 mg/dL)	46.42 ± 7.04	49.15 ± 4.64	0.53
	Creatinine (0.05 to 0.65 mg/dL)	0.28 ± 0.03	0.29 ± 0.04	0.54
	Sodium (135.0 to 146.0 mmol/L)	144.47 ± 1.74	144.27 ± 4.09	0.52
	Potassium (4.0 to 5.9 mmol/L)	6.487 ± 0.55	7.87 ± 0.98	0.48
	Chloride (96.0 to 107.0 mmol/L)	103.63 ± 1.62	99.66 ± 3.12	0.60
	Calcium (5.3 to 11.6 mg/dL)	10.70 ± 0.63	10.99 ± 0.75	0.73

Liver function	ALT (20 to 61 U/L)	59.30 ± 10.89	78.2 ± 12.29	0.12
	Phosphatase (16 to 302 U/L)	214.90 ± 54.03	300.30 ± 91.81	0.08
	AST (39.0 to 111.0 U/L)	123.30 ± 14.79	209.00 ± 71.43	0.16
	GGT (0 to 6 U/L)	4.20 ± 0.63	4.40 ± 0.97	0.70
	Bilirubin (0.1 to 0.7 mg/dL)	0.34 ± 0.14	0.54 ± 0.10	0.61
	Cholesterol (20 to 92 mg/dL)	5.92 ± 0.43	6.28 ± 0.42	0.70
	Triglycerides (27 to 108 mg/dL)	111.28 ± 46.38	116.65 ± 25.88	0.24

Erythrogram	Erythrocytes (5.1 to 7.9 millions/mm^3^)	6.06 ± 0.63	6.06 ± 0.75	0.76
	Hemoglobin (10.0 to 17.4 g/dL)	13.36 ± 1.05	12.98 ± 1.13	0.45
	Hematocrit (33.0 to 50.0%)	42.16 ± 2.74	40.80 ± 3.05	0.88
	MVC (57.8 to 66.5 u3)	64.94 ± 2.40	67.83 ± 5.95	0.87
	MHC (17.1 to 23.5 pg)	20.54 ± 0.67	21.55 ± 1.45	0.49
	MCHC (29.0 to 37.0 g/dL)	31.68 ± 1.04	31.85 ± 2.07	0.33
	Total protein (5.4 to 8.5 g/dL)	5.92 ± 0.33	6.10 ± 0.45	0.38

Leukogram	Leukocytes (5.2 to 12.5 thousands/mm^3^)	4.48 ± 1.15	5.95 ± 2.01	0.33
	Neutrophil (20 to 75%)	55.60 ± 17.10	63.67 ± 7.89	0.46
	Eosinophil (1 to 4%)	0.00 ± 0.00	0.17 ± 0.41	0.19
	Lymphocytes (30 to 85%)	41.80 ± 18.05	33.00 ± 8.15	0.30
	Monocytes (1 to 4%)	2.60 ± 1.14	3.17 ± 1.47	0.11
	Platelets (250 to 650 thousands/mm^3^)	418.60 ± 102.50	473.67 ± 76.62	0.52

Coagulogram	PT (7.2 to 7.8 seconds)	7.44 ± 0.32	7.35 ± 0.26	0.44
	PTT (35 seconds)	21.38 ± 1.09	22.73 ± 2.23	0.38

N: number of animals in the group; data expressed as mean ± standard deviation. ∗Statistically significant difference *p* < 0.05.

**Table 3:** Effects of P2Et extract on biochemical and hematological parameters in rabbits following 28 days of administration.

	Parameters and reference values	Group		*p* value
		Test (N = 5)	Control (N = 6)	
Kidney function	Urea (13.0 to 52.0 mg/dL)	42.32 ± 11.91	43.68 ± 6.98	0.66
	Creatinine (0.80 to 1.80 mg/dL)	1.34 ± 0.46	1.19 ± 0.33	0.91
	Sodium (138.0 to 148.0 mmol/L)	142.48 ± 1.18	144.35 ± 3.76	0.93
	Potassium (3.3 to 6.9 mmol/L)	4.81 ± 0.46	4.78 ± 0.47	0.55
	Chloride (92.0 to 112.0 mmol/L)	99.64 ± 2.55	98.38 ± 4.56	0.58
	Calcium (5.6 to 12.0 mg/dL)	12.86 ± 0.32	12.88 ± 0.41	0.93

Liver function	ALT (31 to 60 U/L)	162.00 ± 111.60	108.17 ± 21.21	0.62
	Phosphatase (90 to 145 U/L)	145.00 ± 32.52	137.33 ± 44.53	0.50
	AST (42.0 to 98.0 U/L)	103.80 ± 95.05	95.50 ± 53.78	0.65
	GGT (4 to 12 U/L)	6.20 ± 2.49	6.00 ± 1.67	0.81
	Bilirubin (0.30 to 0.80 mg/dL)	0.44 ± 0.07	0.53 ± 0.10	0.23
	Cholesterol (35 to 60 mg/dL)	44.00 ± 0.00	43.67 ± 0.52	0.26
	Triglycerides (124 to 156 mg/dL)	108.34 ± 75.81	76.17 ± 37.28	0.81

Erythrogram	Erythrocytes (5.1 to 7.9 millions/mm^3^)	6.06 ± 0.63	6.06 ± 0.75	0.76
	Hemoglobin (10.0 to 17.4 g/dL)	13.36 ± 1.05	12.98 ± 1.13	0.45
	Hematocrit (33.0 to 50.0%)	42.16 ± 2.74	40.80 ± 3.05	0.88
	MVC (57.8 to 66.5 u3)	64.94 ± 2.40	67.83 ± 5.95	0.87
	MHC (17,1 to 23,5 pg)	20.54 ± 0.67	21.55 ± 1.45	0.49
	MCHC (29,0 to 37,0 g/dL)	31.68 ± 1.04	31.85 ± 2.07	0.33
	Total protein (5.4 to 8.5 g/dL)	5.92 ± 0.33	6.10 ± 0.45	0.38

Leukogram	Leukocytes (5.2 to 12.5 thousands/mm^3^)	4.48 ± 1.15	5.95 ± 2.01	0.33
	Neutrophil (20 to 75%)	55.60 ± 17.10	63.67 ± 7.89	0.46
	Eosinophil (1 to 4%)	0.00 ± 0.00	0.17 ± 0.41	0.19
	Lymphocytes (30 to 85%)	41.80 ± 18.05	33.00 ± 8.15	0.30
	Monocytes (1 to 4%)	2.60 ± 1.14	3.17 ± 1.47	0.11
	Platelets (250 to 650 thousands/mm^3^)	418.60 ± 102.50	473.67 ± 76.62	0.52

Coagulogram	PT (7.2 to 7.8 seconds)	7.44 ± 0.32	7.35 ± 0.26	0.44
	PTT (35 seconds)	21.38 ± 1.09	22.73 ± 2.23	0.38

N: number of animals in the group, data expressed as mean ± standard deviation. ^*∗*^Statistically significant difference *p* < 0.05.

**Table 4:** Baseline characteristics in healthy volunteers.

	Level 1 600 mg/d, *n* = 7
N (%)	7 (100)
Male	4 (57.1)
Female	3 (42.9)
Age (years, mean ± SD)	33.3 (7.76)
Weight (kg, mean ± SD)	62.6 (13.0)
Height (mt, mean ± SD)	1.68 (0.05)
Systolic blood pressure (Hg/mm, mean ± SD)	107 (8.41)
Diastolic blood pressure (Hg/mm, mean ± SD)	74.3 (7.87)
Heart rate (beats per minute, mean ± SD)	75.7 (13.5)

**Table 5:** Incidence of adverse events in level 1 dosing.

	P2Et 600 mg/d	
	*n*	Events (%)
Epigastric pain	3	7 (16.7)
Myalgia	2	3 (7.1)
Retching	3	3 (7.1)
Adynamia	2	2 (4.8)
Asthenia	2	2 (4.8)
Constipation	2	2 (4.8)
Odynophagia	2	2 (4.8)
Reflux	2	2 (4.8)
Acute nasopharyngitis	2	2 (4.8)
Increase AST	1	1 (2.4)
Increase bowel habit frequency	1	1 (2.4)
Increase abdominal bowel sounds	1	1 (2.4)
Cervical pain	1	1 (2.4)
Nail cyanosis at the level of the upper limbs not associated with paresthesias	1	1 (2.4)
Soft stool	1	1 (2.4)
Abdominal distention	1	1 (2.4)
Flatulence	1	1 (2.4)
Hypogammaglobulinemia	1	1 (2.4)
Hyporexia	1	1 (2.4)
Leukopenia	1	1 (2.4)
Neutropenia	1	1 (2.4)
Oliguria	1	1 (2.4)
Polydipsia	1	1 (2.4)
Feeling of fullness	1	1 (2.4)
Sensation of emptiness in epigastrium	1	1 (2.4)
Cough	1	1 (2.4)

AST, aspartate aminotransferase.

**Table 6:** Immunophenotype in healthy volunteers in baseline, final visit, and percentage of change.

	Extract P2Et 600 mg/d			Normal range 1
	Baseline visit	Final visit	% change	
T lymphocyte cells				
CD3^+^/CD5^+^/CD45^+^ (%)	21.9 [16.1, 29.6]	22.6 [20.2, 29.6]	23.9 [−7.79, 40.4]	55–74
CD3^+^/CD5^+^/CD45^+^ (cel/*μ*l)	1120 [1030, 1690]	1150 [1060, 1530]	7.50 [−33.6, 31.6]	850–1850
CD3^+^/CD4^+^ (%)	11.0 [10.0, 12.8]	13.2 [12.0, 15.0]	27.0 [−3.91, 32.0]	27–41
CD3^+^/CD4^+^ (cel/*μ*l)	613 [545, 795]	643 [530, 943]	16.5 [−19.2, 25.7]	440–960
CD3^+^/CD8^+^ (%)	9.10 [4.50, 17.1]	9.00 [6.00, 14.4]	29.3 [−15.8, 54.0]	18–34
CD3^+^/CD8^+^ (cel/*μ*l)	432 [310, 974]	482 [374, 651]	−1.63 [−44.0, 41.6]	300–820
CD4^+^/CD8^+^ (%)	0.30 [0.10, 1.50]	0.20 [0.10, 1.00]	40.0 [−33.3, 100]	
CD4^+^/CD8^+^ (cel/*μ*l)	17.0 [5.00, 69.0]	11.0 [5.00, 66.0]	25.0 [−52.9, 83.3]	
CD4−/CD8− (%)	1.00 [0.30, 1.60]	1.10 [0.30, 2.20]	33.3 [−25.0, 71.4]	
CD4−/CD8− (cel/*μ*l)	60.0 [20.0, 88.0]	52.0 [16.0, 117]	−8.75 [−44.1, 60.	
CD4/CD8 relation	1.25 [0.600, 2.40]	1.30 [1.00, 2.20]	−4.17 [−15.0, 66.7]	1–3.6
NK cells				
CD3−/CD56^+^/CD45^+^ (%)	2.10 [1.50, 9.90]	3.00 [1.30, 5.80]	0.00 [−55.6, 63.6]	F: 2.5–26.7
				M: 4–46.6
CD3−/CD56^+^/CD45^+^ (cel/*μ*l)	137 [82.9, 541]	177 [61.3, 236]	−5.88 [−59.5, 114]	F: 44.9–552.7
				M: 78.1–974.1
B lymphocyte cells				
CD19^+^/CD20/CD38^+^/CD45^+^ (%)	2.80 [1.90, 6.40]	2.60 [2.10, 10.0]	18.4 [−40.5, 56.3]	6–19
CD19^+^/CD20/CD38^+^/CD45^+^(cel/*μ*l)	172 [105, 302]	165 [83.2, 443]	10.4 [−60.5, 47.1]	100–500
sLambda^+^ (%)	1.20 [1.00, 2.40]	1.20 [0.90, 3.80]	20.0 [−44.4, 58.3]	
sLambda^+^ (cel/*μ*l)	75.7 [55.3, 119]	67.9 [35.8, 168]	6.21 [−65.1, 49.1]	
sKappa^+^ (%)	1.60 [0.90, 4.00]	1.50 [1.10, 6.20]	13.0 [−36.8, 55.0]	
sKappa^+^ (cel/*μ*l)	96.4 [49.7, 188]	97.1 [45.4, 274]	7.44 [−58.0, 46.0]	
Granulocytes CD45^+^ (%)	63.2 [48.5, 69.8]	59.7 [48.3, 65.2]	−9.97 [−19.5, 9.93]	45–70
Granulocytes CD45^+^ (cel/*μ*l)	2970 [2760, 5560]	3070 [1830, 4500]	−13.1 [−49.0, 15.3	2100–6100
Monocytes CD45^+^/CD38^+^/CD4^+^ (%)	6.10 [3.40, 7.80]	6.00 [3.70, 8.80]	−5.71 [−25.6, 76.5]	0–10
Monocytes CD45^+^/CD38^+^/CD4^+^(cel/*μ*l)	358 [160, 549]	273 [262, 428]	−19.2 [−23.9, 66.2]	300–900
Eosinophils CD45^+^ (%)	1.60 [0.500, 3.90]	1.50 [0.800, 7.60]	4.35 [−50.0, 140]	0–7
Eosinophils CD45^+^ (cel/*μ*l)	88.4 [39.8, 268]	66.4 [37.7, 556]	10.3 [−48.8, 107]	0–500
Basophils CD45^+^/CD38^+^ (%)	0.750 [0.400, 1.30]	0.700 [0.100, 0.800]	−6.25 [−38.5, 50.0]	0–1
Basophils CD45^+^/CD38^+^ (cel/*μ*l)	36.7 [18.8, 89.5]	32.9 [4.80, 58.5]	−5.52 [−34.6, 17.6]	0–200
Dendritic cells				
Derived monocytes				
CD45^++^/CD38^+^/CD4^+^ (%)	0.300 [0.100, 1.00]	0.650 [0.200, 2.60]	66.7 [−33.3, 500]	0–1
CD45^++^/CD38^+^/CD4^+^ (cel/*μ*l)	13.8 [4.70, 51.2]	38.0 [8.80, 98.4]	34.4 [−31.9, 535]	0–200

Data are in median [minimum, maximum] range; (*n* = 7). W: female; M: male.

## Data Availability

The data used to support the findings of this study are available from the corresponding author on reasonable request.

## Supplementary Materials

Supplementary Materials Table S1. Mean intake of food/water as well as initial and final body weight in rats and rabbits following 28 days of administration.
