# Supplementary material for: Safety Evaluation in Healthy Colombian Volunteers of P2Et Extract Obtained from Caesalpinia spinosa: Design 3+3 Phase I Clinical Trial
Source: Evid Based Complement Alternat Med. 2022 Feb 23;2022:7943001. doi: 10.1155/2022/7943001 (PMC8890855; doi:10.1155/2022/7943001)
Supplement: Supplementary Materials — Table S1. Mean intake of food/water as well as initial and final body weight in rats and rabbits following 28 days of administration. [file 7943001.f1.docx]

**Table S1. Mean intake of food/water as well as initial and final body weight in rats and rabbits following 28 days of administration**

| **Animal** | **Sex** | **Group** | **N** | **Initial weight (g)** | **Final weight (g)** | **Body weight gain* (g)** | **Food (g)** | **Water (mL)** |
| --- | --- | --- | --- | --- | --- | --- | --- | --- |
| **Rats** | **Females** | Test | 5 | 171.40 ± 13.33 | 216.22 ± 7.86 | 44.82 ± 10.47 | 17.58 ± 1.19 | 23.50 ± 3.42 |
|  |  | Control | 5 | 166.31 ± 7.31 | 218.43 ± 1.13 | 52.12 ± 10.36 | 18.80 ± 1.44 | 26.50 ± 5.10 |
|  |  | *p* value | | 0.48 | 0.70 | 0.30 | 0.07 | 0.19 |
|  | **Males** | Test | 5 | 240.60 ± 30.28 | 360.08 ± 0.68 | 119.48 ± 35.53 | 26.03 ± 8.90 | 35.00 ± 3.66 |
|  |  | Control | 5 | 226.10 ± 18.87 | 337.61 ± 5.42 | 111.51 ± 29.17 | 26.15 ± 1.98 | 33.50 ± 6.00 |
|  |  | *p* value | | 0.39 | 0.40 | 0.71 | 0.21 | 0.56 |
| **Rabbits** | **Females** | Test | 2 | 1887 ± 201 | 2170 ± 240** | 390 ± 354** | 130.32 ± 5.16 | 190.0 ± 37 |
|  |  | Control | 3 | 2300 ± 370 | 2850 ± 200 | 550 ± 202 | 175.63 ± 4.84 | 274 ± 62 |
|  |  | *p value* | | 0.52 | 0.96 | 0.15 | 0.01 | 0,003 |
|  | **Males** | Test | 3 | 2043 ± 55 | 2443 ± 115 | 400 ± 75 | 153.38 ± 7.13 | 185 ± 23 |
|  |  | Control | 3 | 2010 ± 53 | 2290 ± 105 | 280 ± 157 | 112.71 ± 5.19 | 166 ± 14 |
|  |  | *p value* | | 0.39 | 0.40 | 0.33 | 0.02 | 0.32 |

N: Number of animals in each group, data expressed as mean ± standard deviation. * Body weight gain = initial weight – final weight. ** N=2
